# Quorum Sensing as Antivirulence Target in Cystic Fibrosis Pathogens

**DOI:** 10.3390/ijms20081838

**Published:** 2019-04-13

**Authors:** Viola Camilla Scoffone, Gabriele Trespidi, Laurent R. Chiarelli, Giulia Barbieri, Silvia Buroni

**Affiliations:** Dipartimento di Biologia e Biotecnologie, Università degli Studi di Pavia, 27100 Pavia, Italy; viola.scoffone@unipv.it (V.C.S.); gabriele.trespidi01@universitadipavia.it (G.T.); laurent.chiarelli@unipv.it (L.R.C.); giulia.barbieri@unipv.it (G.B.)

**Keywords:** quorum sensing, cystic fibrosis, bacterial infections

## Abstract

Cystic fibrosis (CF) is an autosomal recessive genetic disorder which leads to the secretion of a viscous mucus layer on the respiratory epithelium that facilitates colonization by various bacterial pathogens. The problem of drug resistance has been reported for all the species able to colonize the lung of CF patients, so alternative treatments are urgently needed. In this context, a valid approach is to investigate new natural and synthetic molecules for their ability to counteract alternative pathways, such as virulence regulating quorum sensing (QS). In this review we describe the pathogens most commonly associated with CF lung infections: *Staphylococcus aureus*, *Pseudomonas aeruginosa*, species of the *Burkholderia cepacia* complex and the emerging pathogens *Stenotrophomonas maltophilia*, *Haemophilus influenzae* and non-tuberculous Mycobacteria. For each bacterium, the QS system(s) and the molecules targeting the different components of this pathway are described. The amount of investigations published in the last five years clearly indicate the interest and the expectations on antivirulence therapy as an alternative to classical antibiotics.

## 1. Introduction

Cystic fibrosis (CF) is a hereditary, autosomal recessive genetic disease associated with mutations in the gene encoding a membrane-bound chloride channel named cystic fibrosis transmembrane conductance regulator (CFTR) [1]. CFTR dysfunction has been correlated to the secretion of a viscous mucus layer on the respiratory epithelium that facilitates colonization by bacterial pathogens. In addition, a major hallmark of CF clinical phenotype is the dysregulation of innate immune functions, leading to chronic bacterial lung infections and inflammation [2]. Pulmonary disease is the primary cause of reduced life expectancy and death in CF patients [3]. The pathogens most commonly associated with CF lung infections include *Staphylococcus aureus*, *Pseudomonas aeruginosa*, species of the *Burkholderia cepacia* complex as well as emerging pathogens, like *Stenotrophomonas maltophilia*, *Haemophilus influenzae* and non-tuberculous Mycobacteria [4].

Antibiotic therapies are implemented in order to eradicate these infections and slow down the deterioration of pulmonary function. However, by targeting essential bacterial physiological processes, antimicrobial compounds exert a strong selective pressure, facilitating the emergence and spread of resistant isolates [5]. New therapeutic strategies aimed at preventing pathogens from producing virulence factors, rather than killing them, represent an attracting alternative to the use of antimicrobial compounds. In particular, regulatory mechanisms controlling the expression of multiple virulence determinants constitute promising targets for antivirulence therapies [6,7]. 

Quorum sensing (QS) is a cell-to-cell communication process that allows bacteria to collectively modify their pattern of gene expression in response to changes in the cell density and species composition of the microbial community. Processes controlled by QS include the activation of bacterial defense mechanisms, such as the synchronized production of virulence factors (toxins, proteases, immune-evasion factors) and biofilm formation. These responses are activated in response to the extracellular concentration of small soluble autoinducer signal molecules that are produced and secreted by bacteria [8]. Autoinducer molecules comprise a diversity of molecular species such as oligopeptides, furanosyl borate diester (autoinducer-2, AI-2), acylated homoserine lactones (acyl-HSLs), the *Pseudomonas* quinolone signal molecule (PQS, 2-heptyl-3-hydroxy-4-quinolone) and integrated QS signal (IQS, 2-(2-hydroxyphenyl)-thiazole-4-carbaldehyde) as well as the *Burkholderia cepacia* complex fatty acid molecule named diffusible signal factor (BDSF) [9,10,11,12,13]. Interestingly, bacteria usually do not rely on a single signal molecule but different QS-systems acting in parallel or in a hierarchical manner can be found within the same organism [8,14]. As autoinducers concentration increases with bacterial population density, changes in the concentration of autoinducers allow bacteria to monitor their cell numbers. Autoinducers are bound by specific receptors that reside either in the inner membrane or in the cytoplasm. Once a certain threshold of signal concentration is reached, a cascade of signaling events is triggered, leading to the modulation of the expression of hundreds of genes underlying various biological processes related to bacterial physiology, virulence, and biofilm formation [8]. 

QS is one of the most intensively studied targets for antivirulence therapy. As this process allows the concerted regulation of several virulence determinants without being essential for growth, targeting QS allows controlling bacterial pathogenesis while limiting selective survival pressure and emergence of antibiotic resistance [14]. 

Interference with QS systems therefore represents a promising strategy to address the emergence and spread of antibiotic resistance [7]. A great diversity of QS interfering agents has been described. These compounds can be either of natural or synthetic origin and can target different steps of the QS cell-to-cell communication process, by acting as inhibitors or agonists of signal molecule biosynthesis, signal molecule detection, or signal transduction.

Plant-derived compounds have been known since ancient times as having beneficial properties, including antimicrobial activity. Plant-derived secondary metabolites have been widely explored for their ability to inhibit QS. To test the inhibitory activity of natural compounds, different methods have been developed. The ability of phytochemicals to inhibit violacein production in the sensor strain *Chromobacterium violaceum* (CV12472) is a common assay used to evaluate anti-QS activity [15,16]. In *C. violaceum*, synthesis of the violet pigment violacein is regulated by QS in response to the concentrations of the autoinducers C6-AHL and C4-AHL [17]. Since this QS-regulated trait is easily observable by disc diffusion assay, *C. violaceum* is widely used as biosensor strain for screening anti-QS molecules. More specific and targeted screening methods for anti-QS activity include biofilm formation and eradication assays by crystal violet staining [18,19,20], quantification of QS-regulated virulence traits (e.g., pyocyanin production in *P. aeruginosa*, alpha-hemolysin secretion by *S. aureus*, protease production) as well as gene expression analysis of known QS-targets [21,22].

In this review, we will describe the QS systems of the main pathogens associated with CF lung infections and we will provide an overview of the main, so-far developed antivirulence compounds targeting these cell communication and regulatory processes, focusing our attention on papers published in the last five years.

## 2. *Staphylococcus aureus*

*Staphylococcus aureus* is a ubiquitous non-motile Gram-positive coccus, which can be found in the anterior nares and skin of humans. It is an aerobe and a facultative anaerobe bacterium, able to form biofilms, which can cause skin, soft tissue, and respiratory infections, osteomyelitis, endocarditis, and can colonize medical device implants. It can cause bacteraemia in 30–50% of healthy people with chronic nasal carriage [23]. Within two years of the introduction of methicillin in clinical practice, *S. aureus* strains developed resistance through the acquisition of the *mecA* gene, thus being defined as Methicillin Resistant (MRSA) [24]. Treatment of Methicillin Sensitive strains (MSSA) includes the use of fusidic acid in combination with oxacillin or dicloxacillin (or rifampicin in case of penicillin allergy) given for 14 days [25]. Among the currently used drugs to treat MRSA we can find fusidic acid, trimethoprim-sulfamethoxazole, tetracyclines, linezolid, clindamycin, levofloxacin, glycopeptides, rifampin, aminoglycosides, and tigecycline [26]. Newer medications for MRSA include quinopristin/dalfopristin, daptomycin (for skin infections and bacteriaemia, but not for pneumonia), and fosfomycin in combination with tobramycin [26].

### 2.1. S. aureus Infections in cystic fibrosis

In CF patients *S. aureus* is implicated in early lung damage [27], but it is also associated with lower respiratory tract inflammation [28]. Chronic infections include high bacterial density, frequent exacerbations, and inflammation. Co-infection with *Pseudomonas aeruginosa* and *Stenotrophomonas maltophilia* appear to be particular risk markers for more severe lung disease [29], while in the absence of other infections the prognosis is more favorable [30]. MRSA represent a great concern leading to an increased rate of decline in lung function and a high risk of death [31,32]. At the same time, the emergence of small colony variants (SCVs) is associated with worse lung function [33].

*S. aureus* is often the first pathogen isolated in CF children and the most prevalent one during childhood [34]. From adolescence to adulthood the prevalence of *S. aureus* decreases gradually, but a significant percentage of adults harbor the pathogen [34]. *S. aureus* infections prevalence seems to vary from country to country and over time: in the USA it changed from 30% in 1990 to 60% in 2016 [34]. On the contrary, the UK CF Registry shows a reducing proportion of children infected by *S. aureus*, with 16% of 0–3 year-olds and 23.7% of 4–7 year-olds [35], while in 1994 up to 60% of babies included in a randomized trial were positive [36]. Also, the type of strains is different in the USA respect to Europe: in particular, a three-fold greater annual prevalence of MSSA and an eight-fold greater annual prevalence of MRSA was reported in the USA compared to the UK [26].

Although the high prevalence of infections caused by *S. aureus* no international guidelines for the treatment in CF patients exist [37]. As an example, in UK an anti-staphylococcal prophylactic therapy in younger children is used [38], while in other countries the infection is treated only if symptoms occur or if specimens from airway are cultured positive [39].

### 2.2. Quorum Sensing Systems of S. aureus

**Figure 1:**
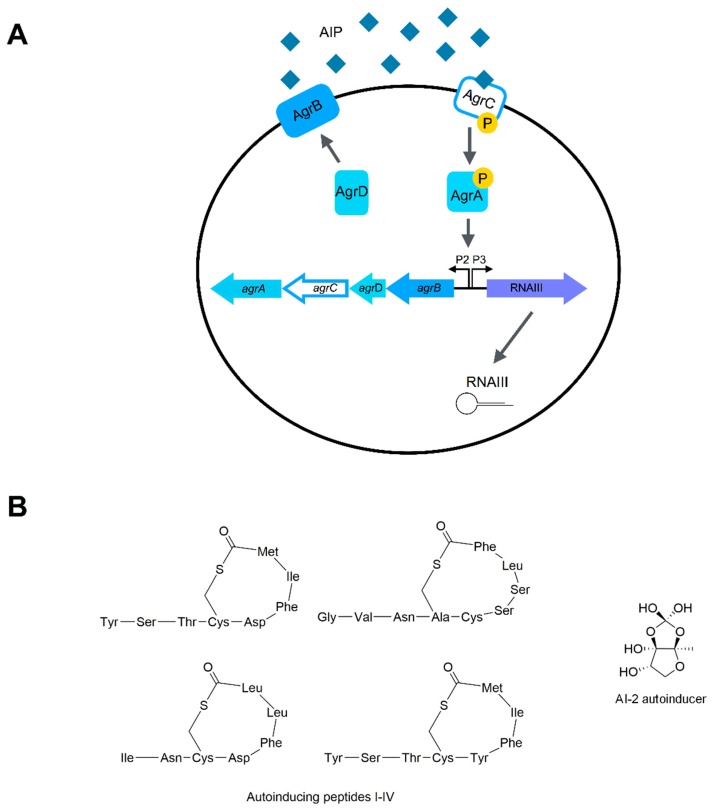
The Agr QS system of *S. aureus*. (**A**) The autoinducing peptide (AIP) is produced by AgrD and exported by AgrB. The two component system AgrC/AgrA is activated by AIP. AgrA binds the P2 and P3 promoter regions activating the *agr* quorum sensing feedback mechanism and RNAIII expression. (**B**) Chemical structures of autoinducing peptides I–IV and autoinducer-2 (AI-2).

Two main QS systems have been described so far in *S. aureus*: the Accessory gene regulator (Agr, Figure 1) and the LuxS systems [40].

#### 2.2.1. The Agr System

The *agr* locus consists of two divergent transcriptional units, RNAII and RNAIII, which are under the control of P2 and P3 promoters, respectively [41]. The RNAII locus contains the genes *agrB*, *agrD*, *agrC* and *agrA* which are transcribed on the complementary strand [42].

The *agrD* gene codes for a peptide which is a precursor of the extracellular AutoInducing Peptide (AIP), the QS signal molecule produced by the Agr system. The AIP harbors a thiolactone ring and an exocyclic N-terminus tail [9].

AgrB is a transmembrane endopeptidase that modifies the thiolactone, cleaves the C-terminal, and exports the AIP [43].

The *agrC* gene encodes a receptor histidine kinase that autophosphorylates and, subsequently, transfers a phosphoryl group to the response regulator AgrA [44]. Then, AgrA activates the P2 promoter region for RNAII, thus leading to an autoinduction, the P3 promoter region for RNAIII [45], but also the transcription of the phenol-soluble modulin psmα and psmβ, a family of staphylococcal peptide toxins [46].

RNAIII contains the gene which encodes the δ-toxin (an exoprotein that lyses eukaryotic host cells), and regulates other genes required for exotoxin secretion, degradative exoenzyme production, and biofilm disassembly [47].

There are four *S. aureus* Agr allelic variants (I to IV) that produce four AIPs which differ in a few amino acid residues (Figure 1B). AIPs function as QS activator in the *S. aureus* cells that produce them, while they generally inhibit QS in *S. aureus* strains that produce different AIPs [47]. Agr groups have been also correlated to specific biotypes: for example, most toxic shock syndrome strains belong to Agr group III, while vancomycin susceptible strains belong to group II and host exfoliation, producing strains belonging to group IV [48].

It has been shown that Agr is inactive during biofilm formation, mainly related to the increased expression of adhesins. In contrast, Agr is active in the detachment process, facilitating bacterial dissemination to other sites [49]. On the other hand, the upregulation of Agr enhances the production of virulence factors important for the progression of many staphylococcal diseases, including pneumonia [50], endocarditis [51], septic arthritis, and osteomyelitis [52,53], and skin and soft tissue infections [54].

#### 2.2.2. The LuxS System

The LuxS system employs the AI-2 autoinducer, a furanosyl borate diester [55]. It regulates the capsule synthesis, biofilm formation (through the *icaR* locus), antibiotic susceptibility, and virulence [56,57]. However, the role of the LuxS system in *S. aureus* QS is not completely clear, as it is involved in metabolism, nor have AI-2 receptors been described so far [58].

### 2.3. Molecules Targeting QS in S. aureus

**Figure 2:**
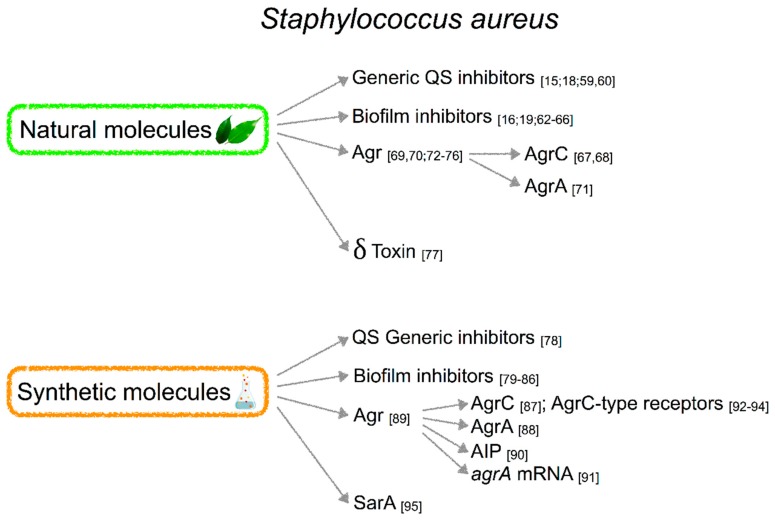
Targets of natural and synthetic molecules active against *S. aureus* quorum sensing.

Molecules targeting QS in *S. aureus* can be classified into natural and synthetic products. Among natural molecules, Agr, generic QS, biofilm, δ toxin, and PT13 inhibitors have been described. Among synthetic products we can enumerate Agr, generic QS, biofilm and SarA inhibitors (Figure 2).

#### 2.3.1. Natural Molecules

**Figure 3:**
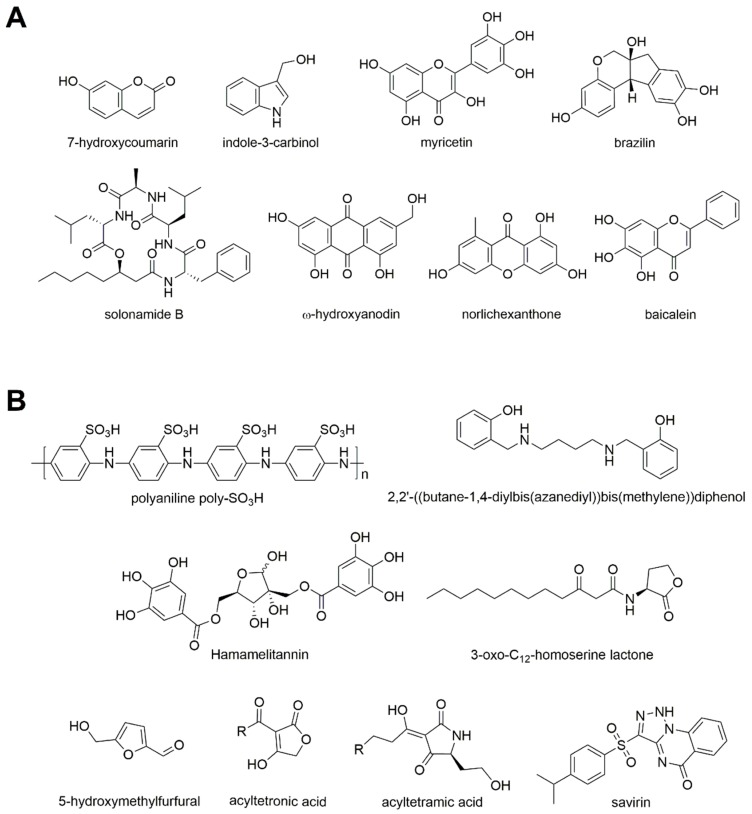
Chemical structures of natural (**A**) and synthetic (**B**) molecules active against *S. aureus* QS systems.

The chemical structure of natural molecules inhibiting QS of *S. aureus* is reported in Figure 3A.

##### Generic QS Inhibitors

Since plants are considered the greatest source for obtaining new antimicrobials, Monte and collaborators explored the antimicrobial activity of four phytochemicals, finding that 7-hydroxycoumarin and indole-3-carbinol affected the motility and QS activity of *S. aureus* [15].

Among generic QS inhibitors of *S. aureus*, an Agr-like peptide from *Clostridium difficile* [59] and myricetin [60] were described. The first one was shown to affect the gene and protein expression profiles of different *S. aureus* strains and inhibited the production of Hla and LukS-PV toxins which are particularly important in *S. aureus* pathogenesis, suggesting its potential use as ‘antipathogenic’ therapy for *S. aureus* infections [59]. Myricetin is a flavonoid contained in fruits, vegetables, tea, berries, and red wine with proven beneficial pharmacological properties [61]. It has been shown to affect both surface and secreted proteins, decreasing the production of several *S. aureus* virulence factors, including adhesion, biofilm formation, hemolysis, and staphyloxanthin production, without interfering with growth, and thus being an alternative multi-target antivirulence candidate [60].

Finally, the alanine rich protein PT13 from *Populus trichocarpa* has been shown to suppress the expression of various QS dependent virulence factors in *S. aureus*, including biofilm-related genes, cell adhesion, and bacterial attachment [18].

##### Biofilm Inhibitors

Mare colostrum, the mare’s first milk, has been shown as a promising source for isolating next-generation antibacterials [16]. It exhibited inhibitory activities against virulence factors produced by *S. aureus*, such as spreading ability, hemolysis, protease, and lipase activities. Moreover, mare colostrum showed a strong inhibitory activity against biofilm formation and eradication [16].

Brazilin is a principal active component of the herbal medicine *Caesalpinia sappan* L [62]. Different studies demonstrated its multiple biological properties, including immune system modulatory, antioxidant, anti-inflammatory, antiplatelet, antihepatotoxicity, and antitumor activities [62], as well as its antimicrobial activity [63]. In 2018, Peng and collaborators used a biofilm model of *S. aureus* to establish in vitro inhibitory effects of brazilin on biofilm formation [19]. Indeed, this molecule was able to inhibit and destroy *S. aureus* biofilm, to reduce the production of the extracellular polymeric matrix and to inhibit the QS system, thus supporting its use as a novel drug and treatment strategy for *S. aureus* biofilm-associated infections [19].

Among natural agents able to inhibit biofilm formation in *S. aureus*, the action of essential oils has been investigated. *Eucalyptus globulus* essential oil, and its main component 1,8-cineole, has been reported to be effective against MRSA [64], while Sharifi et al. [65] investigated the effects of *Thymus daenensis* and *Satureja hortensis* essential oils on some *S. aureus* isolates showing a significant inhibitory effect on biofilm formation.

*S. aureus* expresses several phenol-soluble modulins which are produced in the late growth phase in an *agr* QS dependent manner. They show persister reducing activity which has been associated with lytic activity against bacterial membranes [66]. This way, it has been proposed that these toxins increase the ability of antibiotics to kill persister cells present in the biofilm [66].

##### Agr Inhibitors

Solonamide B is a cyclodepsipeptide isolated from the marine bacterium *Photobacterium halotolerans* that strongly reduces expression of RNAIII, interfering with the binding of *S. aureus* AIPs to sensor histidine kinase AgrC and is the first described natural compound with these characteristics [67]. In 2018, Hansen and collaborators synthesized an array of 27 analogues identifying an analogue resembling solonamide B in amino acid sequence with more potent AgrC inhibitory activity [68].

Manuka honey is another Agr system inhibitor described between 2014 and 2018 [69]. It has been reported as a broad-spectrum antimicrobial agent [70] which is used to treat topical wounds. In order to better understand its mode of action, Jenkins and collaborators performed proteomic and genomic analysis to investigate its effects on MRSA strains [69]. The *agr* gene was found among the genes with decreased expression, thus the authors concluded that a decreased expression of virulence genes will impact MRSA pathogenicity.

ω-hydroxyanodin from *Penicillium restrictum* is a polyhydroxyanthraquinone. It has been shown to prevent *agr* signaling by all four *S. aureus agr* alleles [71]. In particular, it inhibited QS by direct binding to AgrA, thus preventing its interaction with the *agr* P2 promoter. Its efficacy has been demonstrated in a mouse model of *S. aureus* infection where it decreased dermonecrosis in association with enhanced bacterial clearance and reductions in inflammatory cytokine transcription and expression at the site of infection [71].

*Hamigera ingelheimensis*, a metabolically prolific Eurotiales, was observed to be producing an unknown congener, designated as avellanin C [72]. Its chemical structure was determined and its ability to inhibit *S. aureus* QS was reported in 2015 by measuring the decrease in luminescence intensity from a *S. aureus* transformant that carried a plasmid encoding luciferase gene under *agr* P3 promoter upon a treatment with 0.5–200 μM of compound [72].

Also, the QS inhibitory activity of chestnut leaf extracts, which are rich in oleanene and ursene derivatives, has been assessed against all *S. aureus agr* alleles, suggesting a role for non-biocide inhibitors of virulence in future antibiotic therapies [73].

Norlichexanthone is a small non-reduced tricyclic polyketide produced by fungi and lichens. It is able to reduce expression of α-hemolysin and RNAIII thus lowering *S*. *aureus* toxicity towards human neutrophils and reducing its ability to form biofilms [74].

In 2016 Chen and collaborators showed that baicalein treatment reduced staphylococcal enterotoxin A and α-hemolysin levels downregulating the QS regulators *agrA*, RNAIII, and *sarA*, and gene expression of *ica* [75]. Moreover, it was able to inhibit *S. aureus* biofilm formation, to destroy biofilms, and to increase the permeability of vancomycin, supporting its use as a novel drug candidate [75].

Finally, ajoene is a sulfur-rich molecule from garlic which has been shown to reduce expression of key QS regulated virulence factors in *S. aureus* lowering RNAIII expression and, in turn, of hemolysins and proteases [76].

##### δ-Toxin Production Inhibitors

Recently, Khan and collaborators selected nine plants from the Sudhnoti district of Pakistan used in the ethnopharmacological tradition for the treatment of infectious and inflammatory diseases to check their activity against *S. aureus* and other bacteria [77]. Some of the extracts exhibited significant QS inhibition in a reporter strain for *S. aureus agr* I and one of them also for *agr* I–III with a significant drop in δ-toxin production.

#### 2.3.2. Synthetic Molecules

The chemical structure of synthetic molecules inhibiting QS of *S. aureus* is reported in Figure 3B.

##### Generic QS Inhibitors

Three biaryl hydroxyketone compounds showed efficacy in MRSA-infected animal models and combination therapy with cephalothin or nafcillin revealed survival benefits [78]. These data suggested a possible employment of obsolete antibiotic therapies in combination with these novel quorum-quenching agents.

##### Biofilm Inhibitors

Sub-inhibitory concentrations of azithromycin have been shown to decrease the biofilm formation in MRSA in a dose-dependent manner [79].

In 2015, Gizdavic and collaborators showed that functionalized polyanilines significantly disrupted and killed bacterial cells present in pre-established forty-eight-hour static biofilms of *S. aureus* [80].

An acyclic diamine, (2,20-((butane-1,4-diylbis(azanediyl)bis(methylene))diphenol) showed good antimicrobial and antibiofilm activity, being capable of reducing the virulence factors expression. Moreover, confocal laser scanning microscope analysis showed biofilm reduction as well as bacterial killing, suggesting its role as lead compound for further studies in alternative therapeutic approaches [81].

Many studies have been performed also on Hamamelitannin, shown to increase *S. aureus* biofilm susceptibility towards vancomycin through the TraP receptor by affecting cell wall synthesis and extracellular DNA release [82]. Vermote and collaborators synthesized many derivatives [83,84] which resulted in the identification of an analogue that increases the susceptibility of *S. aureus* towards antibiotics in vitro, in *Caenorhabditis elegans*, and in a mouse mammary gland infection model, without showing cytotoxicity [85].

Also, 5-hydroxymethylfurfural has been shown to reduce the ability of *S. aureus* to form a biofilm up to 82% [86], while silver and ruthenium nanoparticles caused a significant reduction in biofilm formation (46%) of a clinical MRSA isolate. Indeed, RNA sequencing demonstrated down-regulation of many biofilm-associated genes and of genes related to virulence [86].

##### Agr Inhibitors

Among synthetic inhibitors of the *S. aureus* Agr system, two kind of small molecules were synthesized: a series of 3-oxo-C12-HSL, tetramic acid, and tetronic acid analogues [87] and Savirin [88]. The former are noncompetitive inhibitors of the AIP activated AgrC receptor and in vivo reduced nasal cell colonization and arthritis [87]. Savirin (*S. aureus* virulence inhibitor) hits the transcriptional regulator AgrA, preventing virulence gene upregulation. Also, savirin showed efficacy in murine skin infection models, abating tissue injury and promoting clearance [88].

In 2014 O’Rourke and collaborators identified a set of peptides displayed on virus-like-particles that bound with high specificity to AP4-24H11. Immunization with a subset of these particles protected against pathogenicity in a mouse model of *S. aureus*, paving the way for the development of a mimotope vaccine [89].

Serum lipoproteins are dual purpose molecules that contribute to both cholesterol homeostasis and host innate defense. The apolipoprotein B100 (apoB100) prevents *agr* activation by binding and sequestering AIP. ApoB48, the N-terminal 2152 amino acids of apoB100, has been shown to antagonize *S. aureus* QS. Since they are produced by enterocytes in the form of chylomicrons, these data suggested a previously unrecognized role for chylomicrons and enterocytes in the host innate defense against *S. aureus* QS-mediated pathogenesis [90].

Peptide-conjugated locked nucleic acids targeting *agrA* mRNA were developed to inhibit *agr* activity and arrest the pathogenicity of MRSA strains. They were shown to inhibit the expression of virulence genes that are upregulated by Agr and showed high levels of protection in a mouse skin infection model [91].

Analogues of a native AIP-III signal able to inhibit AgrC-type QS receptors and attenuate virulence phenotypes in *S. aureus* [92], as well as AIP-II peptidomimetics with a conserved hydrophobic motif [93] or linear peptide-like molecules [94] suggested that the AIP scaffold is amenable to structural mimicry for the development of synthetic QS inhibitors.

##### SarA Inhibitors

The quorum regulator SarA of *S. aureus* up-regulates the expression of many virulence factors, including biofilm formation. Through an in silico approach, Balamurugan and collaborators synthesized 2-[(Methylamino)methyl]phenol, which showed antibiofilm and antivirulence activity against clinical *S. aureus* strains [95].

## 3. *Pseudomonas aeruginosa*

*P. aeruginosa* is a social, ubiquitous, opportunistic Gram-negative pathogen able to cause infections in many different niches of the human body, such as respiratory and urinary tracts [96]. It is highly invasive, toxigenic and adaptable to different surfaces and tissues. Infections occur frequently in immunocompromised individuals and in particular in cystic fibrosis (CF) patients. In 2017 carbapenem-resistant *P. aeruginosa* has been listed in the highest category of “critical” pathogens with urgent need for new treatments by the World Health Organization (WHO) [97]. *P. aeruginosa* is a member of the large group of the ESKAPE pathogens (*Enterococcus faecium*, *Staphylococcus aureus*, *Klebsiella pneumonia*, *Acinetobacter baumannii*, *Pseudomonas aeruginosa*, and *Enterobacter* species) and it could be considered a “superbug” due to its pathogenesis and transmission. The extensive use of antibiotics increases the development of multidrug-resistant *P. aeruginosa* strains that leads to the failure of the therapies against this bacterium [98]. In this scenario, the identification of new and alternative strategies for prevention and treatment of infection is essential.

### 3.1. P. aeruginosa Infections in cystic fibrosis

Chronic *P. aeruginosa* colonizations in CF patients are recalcitrant to antibiotic treatments and they are associated with loss of lung function, morbidity and mortality [99]. The majority of the CF patients become positive for *P. aeruginosa* infections during the lifespan and it is still one of the major causes of death associated with this genetic disorder [100]. Initially, patients are infected by the nonmucoid strains of *P. aeruginosa*, but during the time mutations could occur in *mucA*, encoding an anti-sigma factor, leading to a switch to the mucoid phenotype characterized by the overproduction of polysaccharide alginate [101].

Nowadays, several treatments have been applied to handle early *P. aeruginosa* infection, such as inhaled antibiotics like colistin and tobramycin [102,103], oral ciprofloxacin [104], or an intravenous combination of an aminoglycoside with a beta-lactam [105]. Nevertheless, there is insufficient information to determine the antibiotic strategy that should be used for early *P. aeruginosa* infections eradication in CF patients [106].

### 3.2. Quorum Sensing Systems of P. aeruginosa

**Figure 4:**
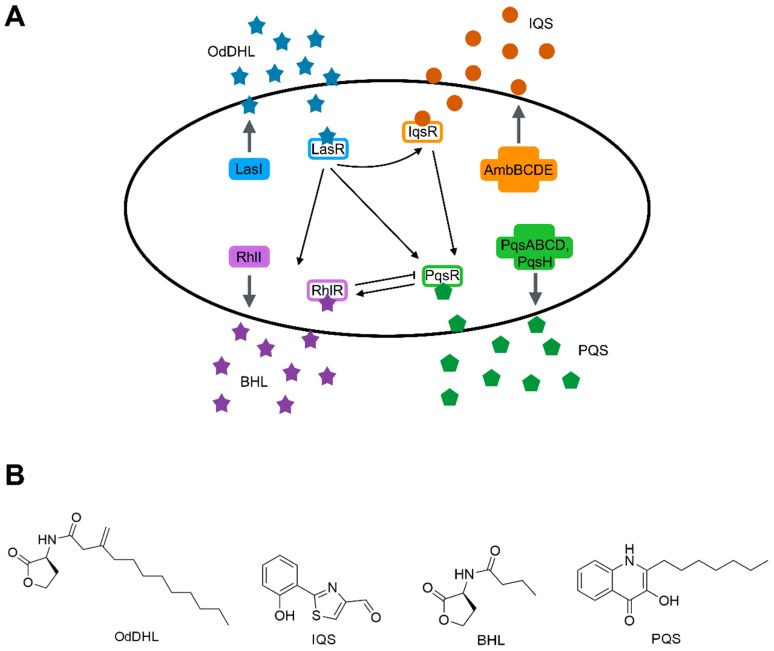
The QS systems of *P. aeruginosa*, Las, Iqs, Rhl and Pqs and their interactions. (**A**) OdDHL, *N*-(3-oxododecanoyl) homoserine lactone; IQS, integrating quorum sensing signal; BHL, *N*-butyryl-l-homoserine lactone; PQS, *Pseudomonas* quinolone signal. Arrows indicate positive regulation, T-bars negative regulation. (**B**) Chemical structures of QS signal molecules.

*P. aeruginosa* is one of the model organisms in QS study and its complex QS systems play a key role in virulence. Four QS systems were described in *P. aeruginosa*: LasI/LasR, RhlI/RhlR, Pqs and Iqs (Figure 4A). Their specific signal molecules are *N*-oxododecanoyl-l-homoserine lactone (OdDHL or 3OC_12_-HSL), *N*-butanoyl-l-homoserine lactone (BHL or C_4_-HSL), the *Pseudomonas* quinolone signal (PQS), and the integrated quorum sensing signal (IQS), respectively (Figure 4B). These systems are deeply intertwined and LasI/LasR is at the top of the hierarchical organization. LasI/LasR and RhlI/RhlR are *N*–acylhomoserine lactone (AHL) circuits homolog of LuxI/LuxR and are activated by an increased cell density. Both these systems are represented by a Lux-type synthase and a LuxR-type receptor. The LasI synthase produces the signal molecule OdDHL which is detected by the cytoplasmic receptor LasR. In the second system, the signal molecule BHL is produced by the synthase RhlI and sensed by the receptor RhlR. The two receptors LasR and RhlR are the transcriptional regulators that control the expression of nearly 10% of the *P. aeruginosa* genome (approximately 300 genes) [107]. The third system is the *P. aeruginosa* quinolone signal (PQS) system in which the signal molecule PQS is produced by PqsABCDE and PqsH and detected by the receptor PqsR [108]. The fourth QS system, activated by phosphate and iron starvation, is still under investigation. Its signal molecule is the IQS synthesized by AmbBCDE [109].

Additionally, *P. aeruginosa* produces other 50 AHQs, discovered by LC/MS of the culture supernatant, the majority of which is still uncharacterized [110].

The *las* system is at the top of the signalling hierarchy and, when activated by its molecule OdDHL, induces the transcription of *rhlI/rhlR*, *lasI* and of the other virulence genes [111]. On the other hand, when RhlR is activated by the BHL signal it induces the expression of *rhlI* and of its own regulon. At the same time, the operon *pqsABCDE* is induced by the *las* system and repressed by rhl and it is modulated by the ratio between OdDHL and BHL [112]. The PQS system induces the transcription of *rhlI* and, in turn, of the RhlI/RhlR system [111,113]. The IQS system is controlled by LasI/LasR during growth in rich medium [109]. PQS signal is produced mainly during the late phase of growth, suggesting its prominent role under stressful conditions [113,114].

Among the virulence factors modulated by QS signals there is the LasB elastase controlled by OdDHL and the BHL signals and involved in the degradation of the proteins of the matrix; the pyocyanin, necessary for immune evasion and controlled by all the three QS systems (OdDHL, BHL and PQS); the protease LasA, for the disruption of the epithelial barrier and the alkaline protease, degrading the proteins of the host defence, both controlled by OdDHL. Also, rhamnolipids, inducing necrosis of immune cells, are controlled by BHL and factors that enhance colonization, such as the LecA lectin regulated by PQS system [11]. QS systems control the production of virulence factors necessary to survive during the host invasion in the early and late stages of infection and in *P. aeruginosa* are required for complete virulence in different hosts: nematodes, fruit flies, zebrafish and mice [115]. *P. aeruginosa* strains deficient in QS systems are significantly less virulent and cytotoxic and induce lower level of tissue damage during colonization [116]. Notably, *P. aeruginosa* clinical isolates from chronic infections showed mutations in the gene *lasR* and some of these mutants still have a functional LasR while others have uncoupled the LasI/LasR system from the RhlI/RhlR [117]. Moreover, stress conditions during infection of the host such as phosphate and iron limitation induce virulence factor production through RhlR and IQS activation [109,118]. It has been demonstrated that there is a correlation between the concentration of some QS signal molecules and the level of pulmonary exacerbation [119]. Hence, some studies were focused on the correlation between the QS and the mucoid phenotype, showing that strains with this phenotype have a reduction of 3-oxo-C12-HSL, C4-HSL and AQ-dependent QS systems during the early stationary phase, while in the late stationary phase expression levels were comparable with the wild type strain [120].

All these results showed how QS systems have a key role during *P. aeruginosa* adaptation to the host and environmental changes.

### 3.3. Molecules Targeting QS in P. aeruginosa

**Figure 5:**
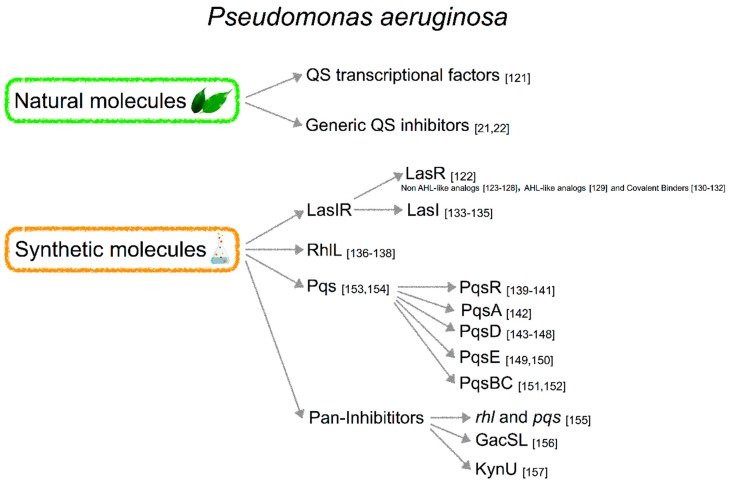
Targets of natural and synthetic molecules active against *P. aeruginosa* quorum sensing.

Also, in this case, both natural and synthetic molecules targeting QS in *P. aeruginosa* have been described (Figure 5).

#### 3.3.1. Natural Products

**Figure 6:**
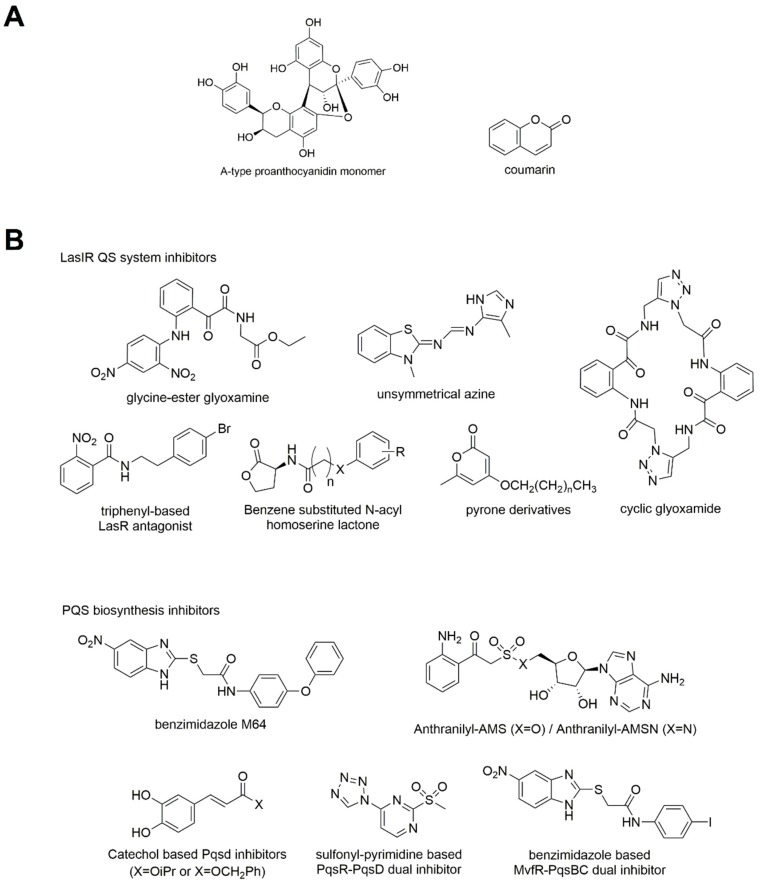
Chemical structures of natural (**A**) and synthetic (**B**) molecules active against *P. aeruginosa* QS systems.

There is a huge amount of literature describing natural compounds active against *P. aeruginosa* QS (Figure 6A). Among these natural products with anti-biofilm activity there is the cranberry extract rich in proanthocyanidins (cerPAC) that acts by decreasing the concentration of virulence factors and protecting *Drosophila melanogaster* from *P. aeruginosa* PA14 fatal infection. *lasIR* and *rhlIR* genes were downregulated and molecular docking studies proposed that CerPAC binds to QS transcriptional regulators [121].

A natural plant phenolic compound, Coumarin, has been described as a QS inhibitor with a strong anti-virulence activity. Coumarin is effective against protease and pyocyanin production and blocks biofilm formation. Furthermore, transcriptome analysis highlighted that several genes involved in *las*, *rhl*, *pqs,* and also IQS systems were downregulated in *P. aeruginosa* PAO1 biofilm treated with Coumarin [21].

The natural compound Baicalin extracted from *Scutellaria baicalensis* inhibits *P. aeruginosa* biofilm formation at sub-MICs concentrations and enhances the activity of bactericidal compounds in vitro. Moreover, it decreases the expression levels of QS-regulatory genes (*lasI*, *lasR*, *rhlI*, *rhlR*, *pqsR* and *pqsA*) and the treatment of *C. elegans* reduces the pathogenesis of *P. aeruginosa* infection and increases the activation of Th1-induced immune response to induce bacterial clearance [22].

#### 3.3.2. Synthetic Molecules

The chemical structure of synthetic molecules inhibiting QS of *P. aeruginosa* is reported in Figure 6B.

##### Inhibitor of LasIR QS System

The LasIR system is an attractive target to interfere with QS and a recent work demonstrates that blocking the LasR receptor prevents the binding of LasR to the target DNA [122]. LasR inhibitors can be classified into three groups: non-AHL-like antagonists, AHL-like antagonists, and covalent binders.

In order to block LasR activity, a possibility is to modify the chemical and enzymatic stability of the molecule. Among the non-AHL-like antagonists there is an indole derivative characterized by low levels of inhibition (65% at 250 µM). More recently, a compound with a glycine ethyl ester branch has been described and tested in the *P. aeruginosa* reporter strain MH602. It blocks the activation of LasR and slightly decreases the pyocyanin production [123]. The same group identified a glyxoamide-based macrocycle able to inhibit the biofilm formation in the reporter strain *P. aeruginosa* MH64 at 250 µM [124].

Among 25 nonsymmetrical azines, another study identified two compounds that inhibit the LasR receptor-based QS system in a plasB-gfpASV-based bioassay and decrease biofilm formation in *P. aeruginosa* [125].

Another hybrid compound was identified using a structure-based scaffold hopping approach combining a triphenyl derivative (already known to agonize LasR) with LasR antagonists producing a more stable molecule. This new compound shows an IC_50_ of 4.8 µM in the *E. coli lasR* reporter strain [126]. Two different patents described two molecules, an *N*-thioacyl-homoserine lactone and a pyrrolidin-2-ol derivative: the first is active against Las, Pqs and Rhl QS system at sub-inhibitory concentration [127], while the second blocks both LasR and RhlR at high concentration (400 µM) [128].

In order to block LasR activity, a family of compounds was designed with an aliphatic tail and a pyrone headgroup; one of these showed the strongest activity at a ligand concentration of 100 µM in the biofilm assay. The interaction between this molecule and LasR was predicted using in silico modeling [129].

In order to interfere with LasR function, many studies developed LasR covalent inhibitors based on the core structure of the ligand. In particular, a previously studied isothiocyanate-based inhibitor, ITC-12, showing covalent non-competitive inhibition at low micromolar concentration, but also able to activate LasR [130], has been modified adding an electronegative halogen. The new compound successfully protects *C. elegans* from *P. aeruginosa* infection [131]. Finally, O’Brien and colleagues synthesized a group of irreversible binders of LasR among which the lead inhibitor is able to decrease *P. aeruginosa* pyocyanine and biofilm production [132].

LasI is another interesting drug target and several studies identified molecules blocking its activity. Unfortunately, there are few examples of LasI inhibitors and the majority described only a putative interaction predicted by docking analysis. Among a group of synthetic and natural compounds, the trans-cinnamaldehyde was identified. This is a strong inhibitor of AHL synthases, decreasing *P. aeruginosa* PAO1 pyocyanin production [133]. Using molecular docking analysis, it has been demonstrated that trans-cinnamaldehyde binds to LasI, interacting with the substrate binding site [133].

The (z)-5-octylidenethiazolidine-2, 4-dione (TZD-C8) is a potent inhibitor of biofilm, swarming motility and QS signal production, and in silico docking studies predicted the affinity of this compound for LasI pocket [134].

In another study on marine *Streptomyces* extracts, it has been shown that some fatty acid lead molecules have synergistic or individual anti-biofilm activity and the most active compounds were successfully docked against the protein LasI [135].

#### 3.3.3. Inhibitors of rhl Quorum Sensing System

A group of synthetic molecules was tested for inhibition of the *Pseudomonas* QS receptor RhlR: among these the most effective compound is an analog of a native autoinducer, the meta-bromo-thiolactone (mBTL), that prevents virulence factor production such as pyocyanin, biofilm formation and protects *C. elegans* and human lung epithelial cells from *P. aeruginosa* infections [136]. The in vivo target of this compound is RhlR and mBTL functions as an agonist of RhlR blocking pyocyanin production down-regulating the Pqs circuit [137]. The same group characterized a family of strong agonists of RhlR that repress the Pqs signal cascade, revealing the Rhl-Pqs cross-talk as a new QS target [138].

#### 3.3.4. Inhibition of PqsR

PqsR could be considered an important target in the development of QSIs. One of the PqsR inhibitors described is an HHQ derivative that once in the cell is converted in a strong PqsR agonist by the synthase PqsH. The molecule was further optimized by introducing a CONH_2_ group which reduced mortality caused by *P. aeruginosa* in an animal model [139].

Another group of PqsR inhibitors was identified using a whole-cell high throughput screen and structure-activity relationship (SAR) analysis: these new molecules block pro-persistence and pro-acute PqsR-dependent signals [140]. These compounds contain the structural backbone of benzamide and a benzimidazole moiety with a thioether bond: they are highly potent with IC_50_ values of 200–350 nM for HHQ, PQS and pyocyanin [140]. The optimization of these molecules resulted in a robust inhibitor called M64 with a significant therapeutic efficacy against acute and persistent infections in mice, also in combination with antibiotic therapy [140]. Moreover, M64 interferes with biofilm formation and potentiates the antibiofilm activity of currently used antibiotics [141].

##### Inhibitors of PQS Biosynthesis

One of the key enzymes of the PQS biosynthesis is the anthranilyl-CoA synthase PqsA, for this reason it is an attractive target for the development of QS inhibitors. Sulfonyladeonsine-based substrate analogs were synthetized: anthranilyl-AMS and anthranilyl-AMSN that decreased HHQ and PQS levels but not pyocyanin production in *P. aeruginosa* [142].

PqsD is involved in the production of the 2-aminobenzoylacetate-CoA, the second step of HHQ biosynthesis. The first PqsD antagonists derived from FabH inhibitors (a homolog of PqsD) and their optimization produced two molecules that compete better for the substrate binding pocket [143]. In another study, urea-based PqsD inhibitors were described and improved, producing compounds with high inhibitory activity (IC_50_ of 0.14 and 0.36 µM) [143]. Unfortunately, their intracellular activity was not evaluable, probably because they were subjected to efflux [144].

PqsD shares some features (size of the active site, catalytic residues) with chalcone synthase (CHS2) of *Medicago sativa.* Starting from these affinities, Allegretta and co-workers evaluated the inhibitory activity of some selected substrates of CHS2 on PqsD. The new inhibitors were characterized by a catechol structure, a saturated linker with at least two carbons and an ester moiety [144]. One of these compounds showed a promising inhibitory activity (IC_50_ of 7.9 µM) and was able to reduce the HHQ production. Surface Plasmon Resonance revealed that this molecule does not bind to the protein active site, but it is near the entrance of the substrate channel [145].

The 2-sulfonylpyrimidines were reported as dual inhibitors targeting the PQS receptor PqsR and the synthase PqsD. Bioisosteric replacement was used to improve their functionality and allowed to obtain a new dual inhibitor with enhanced efficacy [146]. This molecule reduced biofilm formation, pyocyanin and pyoverdine release and restored ciprofloxacin activity. Moreover, it protected the larvae of *Galleria mellonella* from *P. aeruginosa* infections [146].

Two groups of benzamidobenzoic acids were described as RNAP and PqsD inhibitors. Studying the structural modification needed to increase their selectivity against PqsD, Hinsberger and co-workers identified a new molecule which strongly blocks PqsD activity (IC_50_ of 6.2 µM), but without activity against RNAP [147].

Recently, a new strategy to screen PqsD inhibitors in a *E. coli* cellular model system has been described [148]. Through this technique, a covalent inhibitor derived from the anthranilic acid core of the native substrates was identified, which caused a global inhibition of quinolone biosynthesis in *P. aeruginosa* [148].

Another enzyme that plays a central role in the HHQ biosynthesis is the thioesterase PqsE: its functions are not completely elucidated, but it is involved in the regulation of numerous genes coding for biofilm production and virulence determinants [149]. PqsE contributes to the regulation of bacterial virulence producing an alternative ligand that activates RhlR QS receptor in the absence of the 4-HSL. During the elucidation of PqsE function, molecules that bind at the PqsE catalytic site and that inhibit its thioesterase activity were identified [149]. Further investigations are needed because these ligands failed to alter the levels of the PqsE-regulated virulence factor pyocyanin and to influence the interaction with RhlR [150].

The final step of HH biosynthesis involves the heterodimer PqsBC. Taking advantage of a benzamide-benzamidazole (BB) core structure (described as PqsR inhibitor) the first class of inhibitors targeting simultaneously MvfR and PqsBC has been identified [151]. The authors showed that PqsBC inhibition blocks acute virulence behaviours, interfering with the conversion of 2-ABA into the signal molecule HHQ [151]. The dual inhibition allows to decrease acute and chronic virulence factors. Moreover, these molecules have a more potent efficacy against antibiotic tolerance [151].

In another study, the effect of already described Pqs inhibitors was evaluated on PqsBC activity, underlining that these compounds are less effective in the reduction of HHQ levels. Indeed, these compounds affect the distribution of QS molecules and not their production [152].

There are also other compounds described as Pqs inhibitors with unspecific targets. The 4-aminoquinoline derivatives are effective QS and biofilm inhibitors in *P. aeruginosa*, characterized also by a weak bactericidal activity. Among these molecules, compounds interfering with PQS signalling, able to decrease pyocyanin production and biofilm formation were identified [153].

In order to characterize new PqsR antagonists, in silico docking analysis, together with screening with *P. aeruginosa* mCTX::PpqsA-lux chromosomal promoter fusion were performed. The resulting hits blocked alkylquinolone and pyocyanin production in both *P. aeruginosa* PAO1-L and PA14. Among these, one compound, stable in the plasma, reduced biofilm formation and increased the efficacy of tobramycin [154].

#### 3.3.5. “PAN-INHIBITORS” of QS

Previous studies highlighted the efficacy of ajoene against QS in *P. aeruginosa*, hence a screening of in-house compound library identified a sulfuric compound (resembling ajoene) able to block QS. The optimization of this molecule was carried out using SAR and a benzothiazole derivative was the most potent [155]. These derivatives reduced the production of virulence factors (elastase, rhamnolipids and pyocyanin) and decreased *P. aeruginosa* infection in the animal model [155].

Another group identified benzothiazole-based HK inhibitors that alter multiple virulence factors, in particular the compounds called Rilu-4 and Rilu-12 decreased significantly the production of PQS signal molecule, toxins and altered the motility of the bacteria, acting on the functionality of the two-component system GacS/GacA [156].

The products of *Petiveria alliacea, S*-phenyl-l-cysteine sulfoxide, antagonized QS pathways and biofilm formation and *P. aeruginosa* showed a down-regulation of many QS-dependent virulence operons and a misregulation of genes involved in metabolic pathways such as the one of PQS biosynthesis. Indeed, *S*-phenyl-l-cysteine sulfoxide is able to inhibit the KynU enzyme (kynureninase), reducing the PQS production in vivo [157].

## 4. *Burkholderia cepacia*

### 4.1. B. cepacia Infections in cystic fibrosis

*Burkholderia cepacia* complex (Bcc) is a group of 22 closely related Gram-negative bacterial species isolated from soil, water, plants, industrial settings, hospitals and from infected patients [158,159,160,161]. These bacteria have peculiar characteristics, as the ability to degrade toxic xenobiotics and the capacity of promoting the growth of crops, that make them interesting for biotechnological applications in agriculture and industry [162].

Unfortunately, Bcc bacteria are better known from the early 1980s as opportunistic human pathogens which cause persistent and severe infections in CF patient airways, as well as in chronic granulomatous disease or in immunocompromised individuals. The acquisition of these infections, which can occur both from the environment and from other patients, is very dangerous, because they are inherently highly resistant to the majority of the antibiotics used in current clinical treatments [163,164], making the eradication extremely challenging. This wide antibiotic resistance is shared by clinical and environmental *Burkholderia* strains, because of the conservation of the genes coding for the resistance mechanisms [165]. Moreover, in up to 20% of the cases the infection leads to the cepacia syndrome, a lethal necrotizing pneumonia associated with bacteremia [166]. Although all the Bcc species are able to cause an infection in CF patients, *Burkholderia cenocepacia* and *Burkholderia multivorans* are the most common isolates from these patients [4]. *B. cenocepacia* includes the epidemic strains ET-12 and Czeck strain, which spread in the 1990s within people with CF in Canada and Europe, besides the PHDC and the Midwest clone, dominant in United States [167]. These strains are particularly virulent and transmissible and is demonstrated that they possess several genes coding for virulence factors [168]. The expression of these genes is mainly regulated by QS.

### 4.2. Quorum Sensing Systems of B. cepacia

**Figure 7:**
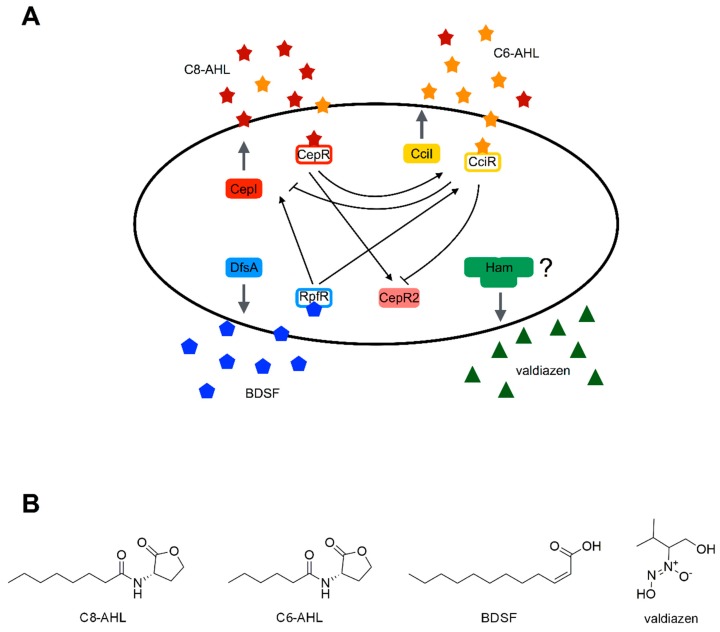
The QS systems of *B. cenocepacia*, Cep, Cci, Rpf and Ham and their interactions. (**A**) The interactions of the Ham system are not completely elucidated. C8-HSL, *N*-octanoyl-homoserine-lactone; C6-HSL, *N*-hexanoyl-homoserine-lactone; BDSF, Burkholderia diffusible signal factor and valdiazen. Arrows indicate positive regulation, T-bars negative regulation. (**B**) Chemical structures of QS signal molecules.

Bcc uses multiple QS systems for the cell-to-cell communication, and while some are common to each species, others are species or strain-specific (Figure 7A). QS in Gram-negative bacteria usually involves the production and sensing of *N*-acyl-homoserine lactone (AHL) molecules synthesized by a LuxI homolog and bound by a transcriptional regulator of the LuxR family (Figure 7B) [169]. This system is represented in all Bcc species by CepIR, which is composed of the synthase CepI that synthesizes *N*-octanoyl-homoserine-lactone (C8-HSL) and, to a minor extent, *N*-hexanoyl-homoserine-lactone (C6-HSL), and CepR that can act both as positive and negative transcriptional regulator [170].

Some epidemic strains of *B. cenocepacia* possess also a pathogenicity island named cenocepacia island (cci) which contains the genes coding for a second AHL-based QS system known as CciIR. Conversely to CepI, CciI produces mainly C6-HSL and a lower amount of C8-HSL, activating the cognate receptor CciR [171].

*B. cenocepacia* has also an orphan LuxR homolog, CepR2, not coupled with any synthase in the genome, indeed its activation is independent from AHLs, even though recently, it has been discovered that its activity is antagonized by C8-HSL [172,173].

Among the Bcc, another AHL-mediated QS system has been characterized only in other two species, *Burkholderia vietnamiensis*, which has the BviIR system that synthesizes C10-HSL [174], and *Burkholderia ambifaria*, which has the CepI2R2 system that mainly produces the hydroxylated AHLs, 3OHC10-HSL and 3OHC12-HSL [175].

The other leading signal molecule used by all Bcc species for cellular communication, is the *Burkholderia* diffusible signal factor (BDSF), a fatty acid molecule similar to the diffusible signal factor originally described in *Xanthomonas* [12,176]. In *B. cenocepacia* BDSF or cis-2-dodecenoic acid is synthesized by the bifunctional crotonase DfsA or RpfF_bc_, the counterpart of RpfF of *Xanthomonas*, [177] and sensed by the soluble receptor RpfR [13,178]. This protein, activated in the presence of BDSF, degrades the c-di-GMP through its phosphodiesterase activity and allows the activation of the global transcriptional regulator GtrR which works in complex with RpfR itself [179].

Furthermore, another sensor protein has been identified, BCAM0227, which controls only a subgroup of genes regulated by BDSF (Figure 7B) [180]. *B. cenocepacia* is able to produce also a 2-heptyl-4(1H)-quinolone (HHQ) molecule, a known signaling factor in *Burkholderia pseudomallei*, but not yet characterized in this species [181]. Besides a modified HHQ, the 4-hydroxy-3-methyl-2-heptenylquinoline (HMAQ-C7:2′) is produced by the other Bcc species *B. ambifaria* [182].

Finally, the last signal molecule discovered in the Bcc has been a diazeniumdiolate compound named valdiazen, which controls the expression of more than 100 genes in *B. cenocepacia* H111 [183]. This molecule is probably the first member of a new wider class of signal compounds in *Burkholderia*. These QS systems communicate with each other through a very complex and regulated network of interactions that is just partially characterized so far, considering that new regulators are discovered periodically and some interactions are not fully understood as yet.

The AHL based QS systems directly interact, and indeed CepR positively regulates the expression of *cepI* by a positive feedback regulation, but also the *cciIR* operon and, at least in *B. cenocepacia* H111, the gene *cepR2*. However, it is also a repressor of its own expression. The *B. cenocepacia* QS regulator, CciR, is instead a transcriptional repressor, negatively regulating the expression of *cepI* by a negative feedback regulation, *cepR2* and its own expression [184]. The orphan regulator CepR2 does not need the presence of AHL to be active and to repress its own expression, as it inhibits the activator CepS, an AraC-type transcription factor, and blocks the transcription in an unusual way [173]. Unlike the other two regulators, CepR2 does not interfere with CepIR and CciIR systems [172].

Until a few years ago, there was no evidence about cross interactions between the two main QS systems of *Burkholderia*, CepIR and BDSF, but today it is assessed that they are part of a bigger regulon. Indeed, it has been demonstrated that BDSF controls the AHL production, activating the RpfR-GtrR complex and directly promoting the expression of the AHL synthase gene cepI [185,186]. Moreover, in the ET-12 strain of *B. cenocepacia* the synthase gene *cciI* is regulated in the same manner [185].

Several other regulators are known to take part in the QS regulation, such as CepS, ShvR, YciR, SuhB, YciL, BCAM1871, AtsR, BCAM1869, BCAM0258, BceR, RqpR, contributing to the complexity of the network [173,187,188,189,190,191,192,193].

QS systems are known to control the expression of next to hundred genes in *Burkholderia*, and several of these are regulated together by more than one system [184,194,195]. In human infections, it has been proved that the bacterium needs active QS systems to infect the lungs of CF patients successfully, because they control also the expression of the genes coding for virulence factors [196]. The virulence factors directly associated with QS are the extracellular zinc metalloprotease ZmpA and ZmpB [12,194], the siderophores ornibactin and pyochelin [184,197], the flagellar motility [180,184], the type III and type VI secretion systems [180,184], the biofilm formation [13,198], the LysR regulator ShvR [199], the protein BCAM1871 not characterized as yet [187], and the nematocidal protein AidA [172,184]. Therefore, in order to decrease the virulence of these infections and to overcome antibiotic resistance, molecules able to hit the QS regulation have been screened and characterized.

### 4.3. Molecules Targeting QS in B. cenocepacia

**Figure 8:**
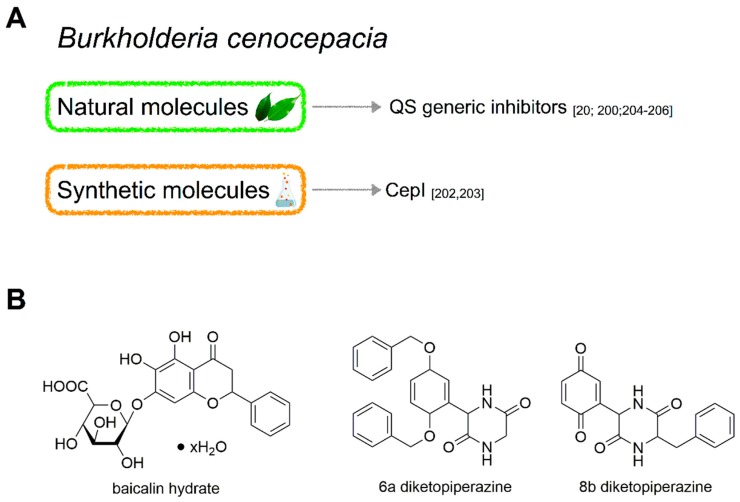
(**A**) Targets of natural and synthetic molecules active against *B. cenocepacia* quorum sensing. (**B**) Chemical structures of natural and synthetic molecules active against *B. cenocepacia* QS systems.

The combination of quorum sensing inhibitors (QSI) with antibiotics is a useful strategy to control the infections caused by Bcc bacteria. Although promising results have been obtained in the last years, only few active molecules in Bcc have been studied, principally because of the low number of studies performed on these bacteria. The molecules characterized as active QSI are both natural compounds and synthetic molecules [20,200,201,202] which are able to interfere with the biofilm formation and in turn to increase the efficacy of the antibiotic treatment (Figure 8A). Moreover, it has been proved that some of these QSI can decrease the virulence in infection models [200,202]. Within them, there are both AHL analogues, that act as agonist or antagonist of the natural AHL [201], and molecules with completely different structures and so different mechanisms of action [202,203]. Recently, a new set of synthetic diketopiperazines has been tested in our laboratory for their ability to inhibit QS, and among them we discovered two molecules with a very interesting activity against CepI of *B. cenocepacia* J2315 [202]. Besides these, the only other molecule further studied in the last years has been the baicalin hydrate [204], a flavonoid initially characterized as QSI for *Pseudomonas aeruginosa* in 2008 by Zeng et al. [205].

#### 4.3.1. Natural Molecules

Baicalin hydrate (BH, Figure 8B) is a polyphenolic molecule belonging to the class of the flavonoids, isolated from the roots of *Scutellaria baicalensis*, and firstly characterized as QSI in a work consisting in the screening of compounds with antibacterial activity of the Traditional Chinese Medicine [205]. Polyphenols are already known to be biofilm and swarming motility inhibitors in *B. cenocepacia* H111 [206]. BH has been tested as QSI on some microorganisms including Bcc bacteria, obtaining very promising results. These studies uncovered its ability to impair the biofilm formation in *B. cenocepacia* and *B. multivorans* [20], and the ability to increase the effect of tobramycin against sessile cells both in vitro and in vivo [200]. The combination of BH and tobramycin results in a significantly reduced mortality after *Burkholderia* infections in *C. elegans* and *G. mellonella* models and in a strongly reduced pulmonary bacterial load in infected mice, compared to the antibiotic treatment alone [200]. Although the potential of this molecule is fully recognized, the mechanism of action of BH remains unclear. BH enhances the bactericidal activity of tobramycin and other aminoglycosides, even though the effect is strain-specific, increasing the formation of reactive oxygen species, by acting on several pathways such as cellular respiration, glucarate metabolism and biosynthesis of putrescine [204]. On the contrary, the QSI action of BH in this case may not be the main mechanism that leads to this synergistic effect as hypothesized by Slachmuylders et al. [204], leaving a question open for further studies.

#### 4.3.2. Synthetic Molecules

2,5-Diketopiperazines (DKPs, Figure 8B) are a class of cyclic dipeptides, isolated mainly from Gram-negative bacteria, but also from Gram-positive bacteria, Archaea and Fungi, which were characterized as agonists of LuxR-type proteins and accordingly a new class of QS molecules and interspecies signals [207,208]. However, this theory is still debated, because of a study which proved that DKPs did not interact directly with TraR, LasR and LuxR [209]. However, being DKPs an established QSI, in order to find CepI inhibitors of *B. cenocepacia* J2315, we have tested ten newly synthesized molecules, designed adding a redox moiety to the DKPs scaffold, against the enzymatic activity of the recombinant purified synthase CepI. We have found two active compounds, named 6a and 8b, classified as non-competitive inhibitor of the synthase. Even though the two molecules did not show any synergistic activity in combination with the currently most used antibiotics against planktonic cells, they were able to significantly reduce the production of siderophores and proteases, and besides to interfere with the biofilm formation [202]. These results have been confirmed also in a *C. elegans* infection model, showing that the two DKPs could protect the nematodes from the infection of *B. cenocepacia* J2315. Moreover, it has been verified that 6a and 8b have a very low toxicity on HeLa cells, making the compounds good candidates for future experimentations in humans [202]. A further study has been performed on the molecule 8b, showing that the effect of the compound on *B. cenocepacia* J2315 is comparable to the deletion of *cepI*, and also that its binding pocket is likely localized close to the predicted *S*-adenosylmethionine binding site of CepI [203], adding information that may be considered in the future to improve its biological activity by chemical modifications.

## 5. Emerging CF Pathogens

### 5.1. Stenotrophomonas maltophilia

#### 5.1.1. *S. maltophilia* Infections in cystic fibrosis

*Stenotrophomonas maltophilia* is a Gram-negative rod which represents an important emerging nosocomial pathogen, responsible for infectious diseases and death, particularly in immunosuppressed or immunocompromised patients or in subjects carrying medical implants [210]. Among the emerging CF pathogens, *S. maltophilia* infection is considerably variable from center to center, ranging from 3–30%, with an increasing in prevalence in this population [211]. In CF patients, infection with *S. maltophilia* has been associated to poor outcomes, decreased lung function, and increased risk of transplantation or death [212].

Nevertheless, the effect of *S. maltophilia* on lung function decline is not clear, and there is no consensus about the management of patients with this infection. For instance, in some cases it is considered a colonizer, and thus no specific treatments are carried out, while in other cases it is treated with specific antibiotics, but with no consensus on the optimal regimen [213].

Anyway, the treatment is very difficult, as *S. maltophilia* is intrinsically resistant to several antimicrobials, and able to acquire new resistances by horizontal gene transfer [214]. Indeed, *S. maltophilia* is usually present in environmental water reservoirs, a highly competitive niche that favors not only the acquisition of resistance genes, but also the establishment of communication networks with the neighboring microorganisms [215].

#### 5.1.2. Quorum Sensing Systems of *S. maltophilia*

The principal QS system of *S. maltophilia* relies on the Diffusible Signal Factor (DSF) cis-11-methyl-2-dodecenoic acid, which regulates bacterial motility, biofilm formation, antibiotic resistance, and virulence [216,217]. Differing from other DSF producing bacteria, such as *B. cenocepacia* and *P. aeruginosa*, in *S. maltophilia,* the genes encoding the QS proteins co-localize in the regulation of pathogenicity factors (Rpf) cluster, and are organized in two adjacent operons convergently transcribed [217]. The first operon encodes the fatty acid ligase RpfB and the synthase RpfF, whereas the second operon contains the genes coding for the sensor kinase RpfC and the cytoplasmic regulator RpfG [216]. The peculiarity of the *S. maltophilia* DSF system is the presence of two variants of the rpf cluster, rpf-1 and rpf-2, which are associated with two RpfC-1 and RpfC-2 variants. Interestingly, the association of these variants is fixed, whereby the strains harboring RpfF-1 necessarily carry the RpfC-1 variants and *vice versa*. The RpfF variants differ in first 108 amino acids, while the RpfC variants display a different number of trans-membrane regions at the N-terminal portion, 10 for RpfC-1 and 5 for RpfC2. To date, the *rpf-1* variant has been detected in the 55.5% of the isolates, while the *rpf-2* in the 44.5% [216]. These two variant strains show differences in DSF synthesis, perception, and in regulation of biological processes. Differently from the *rpf-1* strains, which under standard growth conditions produce DSF, the *rpf-2* needs extra copies of the *rpfF-2* gene or the absence of RpfC-2 to produce DSF [216]. Nonetheless, RpfF-1 and RpfF-2 enzymes have both acyl-ACP dehydratase and thioesterase activity, and catalyze the conversion of (R)-3-hydroxy-11-methyl-dodecanoyl-ACP into DSF [217]. This thioesterase activity is not specific, and the enzymes are able to cleave different medium and long chain acyl-ACP, producing free fatty acids that are released in extracellular environment [217]. Among these fatty acids, the mostly produced in *S. maltophilia* is the 13-methyltetradecanoic acid (iso-15:0). This molecule is synthetized by the biosynthetic pathway of the DSF, suggesting a connection between DSF and membrane synthesis. Indeed, iso-15:0 modulates the DSF in *rpf-1* strains, being sensed by RpfC-1, which thus releases RpfF-1 that can start DSF synthesis [217]. By contrast, in *rpf-2* strains the 5-transmembrane sensor RpfC-2 does not have promiscuous perception, which leads to the repression of RpfF-2 also in the presence of iso-15:0 or other fatty acids. Indeed, the RpfF-2 RpfC-2 complex can dissociate only upon sensing the DSF itself. Thus, in *rpf-2* strains the DSF production is triggered by the presence of exogenous DSF [217].

Interspecies communication through DSF signal molecules is a quite common phenomenon also for *S. maltophilia.* For instance, DSF produced by *S. maltophilia* was found to influence *P. aeruginosa*, particularly regarding biofilm formation, antibiotic resistance, virulence and persistence in lungs of CF patients [218]. Moreover, although *S. maltophilia* has been shown not to produce AHLs, it has been found that it responds to AHL signal molecules produced by *P. aeruginosa* [219]. Indeed, in the *S. maltophilia* genome there are 15 putative LuxR that lack the cognate LuxI, and are widely spread throughout bacteria [220]. Among them, the SmoR, containing the typical N-terminal AHL-binding domain and the C-terminal helix-turn-helix DNA-binding domain, was demonstrated to bind in vitro oxo-C8- homoserine lactone. Moreover, *S. maltophilia* swarming motility was found to be strongly stimulated in the presence of a *P. aeruginosa* supernatant containing high levels of AHLs, indicating that SmoR senses the AHL signals of neighboring bacteria [219]. By contrast, the strain of *S. maltophilia* BJ01 was found to produce a compound (cis-9-octadecenoic acid) which possesses quorum quenching activity and which is able to inhibit biofilm formation of *P. aeruginosa* [221].

#### 5.1.3. Molecules Targeting QS in *S. maltophilia*

Despite the increasing incidence of multi-resistant *S. maltophilia* clinical isolates, and the potential of quenching DSF communication as a promising therapeutic approach, no active compounds, nor QS molecules degrading enzymes, have been reported until now.

### 5.2. Haemophilus Influenzae

#### 5.2.1. *H. influenzae* Infections in cystic fibrosis

Nontypeable *H. influenzae* (NTHi) is a common commensal of the upper airways, which can cause different infections, such as otitis media, bronchitis, sinusitis, and pneumonia. Moreover, chronic infection can occur in patients with diseases of lower respiratory tracts, including chronic obstructive pulmonary disease, bronchiectasis and CF [222]. In particular, NTHi is the most common colonizer of the airways in infants with CF [4]. NTHi is indeed recovered from about 20% of children under the age of 2 years and has a peak of prevalence at 30% in children 2 to 5 years old, decreasing to less than 10% in adults [34]. Furthermore, about 80% of children with *P. aeruginosa* had previous infections with *S. aureus* or NTHi. NTHi has been found involved in chronic infections and exacerbations of CF lung disease, and associated with lower lung function impairment in infants [223].

NTHi is able to produce biofilm, also in the lower airway of CF patients [224]. Similarly, to several pathogens, within biofilm it is intrinsically resistant to antibiotics, and has been demonstrated to be able to persist during infection in multicellular biofilm communities [225]. Moreover, it has been found that low concentrations of different antibiotics stimulate biofilm formation [226].

#### 5.2.2. Quorum Sensing Systems of *H. influenzae*

Biofilm regulation in NTHi is mainly mediated by the autoinducer-2 (AI-2) QS signal that relies on the LuxS/RbsB system [227] and is widely distributed among different bacteria [169]. Interestingly, AI-2 signaling can have different roles among different species, promoting the formation and maturation of the biofilm, or the biofilm dispersal and cells release [228].

In NTHi, AI-2 promotes biofilm formation and prevents its dispersal during the maturation process. Indeed, it has been shown that if the expression of *luxS* is interrupted, thus decreasing the production of AI-2, biofilm dispersal occurs, suggesting a role of AI-2 also in the regulation of the lipooligosaccharides that are known to mediate adherence [229]. In this context, the recent discovery that the levels of AI-2 regulate the expression of the glycosyltransferase GstA [229] paves the way to new investigations to better define how the bacteria form and persist into biofilms, and to find novel possible antivirulence targets. For instance, the AI-2 receptor RbsB has been suggested as a promising target, since mutations in its gene are associated with alterations of biofilm formation and maturation in an in vivo model of otitis media [227,229]. In addition to the LuxS/RbsB system, biofilm in NTHi is regulated by the two-component histidine kinase QseB/QseC system [230]. To date, little is still known about this system in NTHi that, differently from the well characterized QseBC of *E. coli* and *Salmonella enterica*, does not respond to epinephrine or norepinephrine, but is activated only by ferrous iron or zinc and has been designated as a ferrous-iron-responsive system (FirS) [231].

#### 5.2.3. Molecules Targeting QS in *H. influenzae*

**Figure 9:**
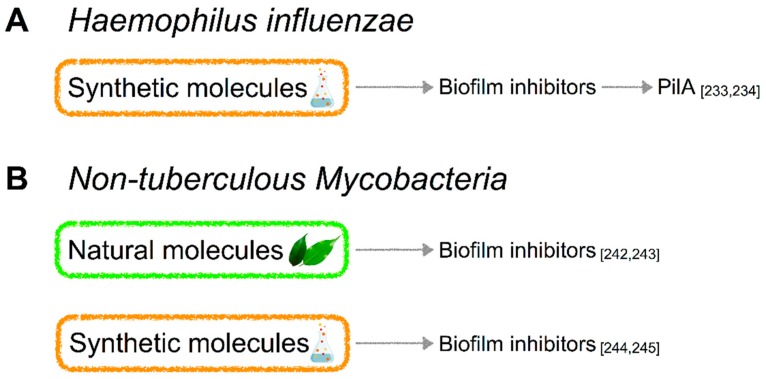
Targets of natural and synthetic molecules active against *Haemophilus influenzae* (**A**) and Non-tuberculous Mycobacteria (**B**) quorum sensing.

A novel approach against NTHi, based on vaccine targeting bacterial adhesive proteins and biofilm mediators, was recently demonstrated to prevent otitis media on a polymicrobial infection model [232]. In particular, it was demonstrated that antisera against the NTHi type IV pili PilA were able to in vitro disrupt and prevent the dual species biofilm formed by NTHi and *Moraxella catarrhalis*. Moreover, the bacteria released from biofilm were significantly more sensitive to different antibiotics [233]. Since NTHi and *M. catarrhalis* have been found together also in the lungs of children with CF [234], this strategy of immunization in combination with antibiotics can provide a novel approach for the treatment of these biofilm-associated infections [233] (Figure 9A).

### 5.3. Non-Tuberculous Mycobacteria (NTM)

#### 5.3.1. NTM Infections in cystic fibrosis

Non-tuberculous mycobacteria (NTM) are microorganisms usually isolated from environmental sources, such as soil and water. NTM can be divided in rapid growers (i.e., *Mycobacterium abscessus* complex, *Mycobacterium fortuitum*), that take less than 7 days to grow, or slow growers (i.e., *Mycobacterium avium* complex, *Mycobacterium kansasii*). Among NTM, some species are associated with human disease, particularly the *M. avium* complex (MAB: including *Mycobacterium avium*, *Mycobacterium intracellulare*, and *Mycobacterium chimaera*) and the *Mycobacterium abscessus* complex (MABSC: that comprises *Mycobacterium abscessus*, *Mycobacterium massiliense*, and *Mycobacterium bolletii*). NTM are considered opportunistic pathogens organisms, particularly prevalent in patients with CF, non-CF bronchiectasis, and in chronic obstructive pulmonary disease [235]. The incidence and prevalence of NTM in CF patients is increasing and highly variable, with a prevalence ranging between 2% and 28% [236]. NTM infections in CF patients, particularly of *M. abscessus*, are associated with an increased morbidity and mortality, and a rapid lung function decline [237]. Moreover, the treatment is particularly challenging due to an intrinsic resistance to several antibiotics, including those commonly used in CF infections [238].

#### 5.3.2. Quorum Sensing Systems of NTM

Most mycobacteria, including NTM such as *M. abscessus* and *M. avium*, are known to form biofilm, suggesting the possibility that these organisms may have QS systems, but experimental validation is still lacking [239]. Nevertheless, bioinformatics analysis demonstrated the presence of homologs of LuxR in *Mycobacterium tuberculosis*, which have been found also in different other mycobacteria [240]. Moreover, the existence of QS in mycobacteria is also indirectly suggested by the fact that their signal transduction phospho-relay cascade uses different di-cyclic or modified nucleotides as second messenger, including c-di-GMP. Indeed, c-di-GMP is ubiquitous in several bacteria, and known to be involved in virulence and biofilm formation [241].

#### 5.3.3. Molecules Targeting QS in NTM

Due to the increasing incidence of NTM infections, together with their intrinsic resistance to several antibiotics, particularly when in biofilms, the research of new compounds with biofilm dispersal activity is fundamental (Figure 9B).

Among the natural products, essential oils, which are secondary plant metabolites, have been suggested as potential antimicrobial and antivirulence compounds [242]. For instance, the essential oil of *Cymbopogon flexuosus* (a plant generally known as lemongrass) was recently demonstrated to have antimicrobial activity against rapidly growing NTM, being also able to efficiently disperse biofilm, as well as to inhibit its formation [243].

On the other hand, Flores and co-workers evaluated the antibiofilm activity of different antibiotics used in the treatment of mycobacterial infections (amikacin, ciprofloxacin, clarithromycin, doxycycline, imipenem and sulfamethoxazole), but none of them was able to significantly prevent biofilm formation or to promote biofilm dispersal [244]. Nevertheless, it was recently found that sulfamethoxazole when in complex with metal ions, particularly in complex with Au, showed a markedly enhanced antibiofilm activity [245]. Moreover, showing these compounds a good safety profile and antibacterial activity, they have been suggested as potential therapeutic agents [245].

Thus, although NTM QS is still very poorly known, these effective antibiofilm compounds demonstrate the potential of this target to develop novel therapeutic molecules.

## 6. Conclusions

In this review we enumerated the principal CF pathogens which cause lung infections: *S. aureus*, *P. aeruginosa*, *B. cepacia* complex bacteria, and the emerging pathogens *S. maltophilia*, *H. influenzae* and non-tuberculous Mycobacteria. As their common peculiarity is the high degree of drug resistance, in the last years a lot of work has been done to find an alternative therapeutic solution: the research on QS inhibitors, able to attenuate the virulence of these strains, has merged as a promising strategy. In this way, both synthetic and natural products were evaluated for their ability to interfere with various components of the QS machinery, although sometimes their target has not been found yet.

In particular, for *S. aureus* we described generic QS inhibitors, both of natural and synthetic origin (Figure 2 and Figure 3). Moreover, among natural molecules, some are able to inhibit biofilm formation, the Agr system at different levels, and the δ toxin; while synthetic compounds hit biofilm formation, the Agr system as well as the QS regulator SarA (Figure 2).

Among *P. aeruginosa* QS inhibitors, we described natural generic and transcriptional factors inhibitors (Figure 5 and Figure 6A). The synthetic molecules reported target all the QS systems of this bacterium: LasIR, RhlIL, and Pqs. Also, Pan-inhibitors have been described (Figure 5 and Figure 6B).

As regarding *B. cepacia* complex, natural generic QS inhibitors have been characterized, while among the synthetic molecules, only CepI inhibitors have been described so far (Figure 8).

Conversely, despite the incidence of multi-resistant *S. maltophilia* clinical isolates, and the potential of quenching DSF communication, no active compounds have been reported until now. The unique report about *H. influenzae* biofilm disruption regards synthetic antisera against the PilA, which render the cells released from biofilm significantly more sensitive to different antibiotics (Figure 9A). Finally, natural essential oils and synthetic sulfamethoxazole in complex with metal ions showed good activity against Non-tuberculous Mycobacteria biofilm (Figure 9B).

The huge amount of literature published in the last five years clearly demonstrates that QS is a good candidate to find alternative therapeutic approaches to face the increasing problem of antibiotic resistance. The compounds described have a great potential as antivirulence molecules, but can also be proposed for combined therapies to improve the activity of existing drugs, paving the way for alternative treatments.

On the other hand, potential challenges for the use of these compounds have been described. These include the possible incurrence of drug resistance [246,247,248], and the limitations of the current animal infection models for QS [246]. Moreover, it seems that, in order to develop truly effective QSI, a better understanding of the virulence and of the behavior of pathogens during infections is needed [249]. Finally, despite QSI having been published in the literature since the 1990’s, none of them is in clinical trial yet, leading to the conclusion that further efforts are needed in order to exploit these new approaches for the treatment of bacterial infections; yet a lot of alternatives are being explored.

## Abbreviations

AHL	Acyl Homoserine Lactone
CF	Cystic Fibrosis
DSF	Diffusible Signal Factor
NTM	Non-tuberculous Mycobacteria
QS	Quorum sensing
QSI	Quorum sensing inhibitor
